# Immunomodulatory activity of a water-soluble polysaccharide extracted from mussel on cyclophosphamide-induced immunosuppressive mice models

**DOI:** 10.1038/s41538-022-00140-8

**Published:** 2022-04-27

**Authors:** Xingwei Xiang, Rui Wang, Lin Chen, Yufeng Chen, Bin Zheng, Shanggui Deng, Shulai Liu, Peilong Sun, Guoxin Shen

**Affiliations:** 1 College of Food Science and Technology, Zhejiang University of Technology, Hangzhou, Zhejiang 310014 People’s Republic of China; 2 Key Laboratory of Marine Fishery Resources Exploitment & Utilization of Zhejiang Province, Hangzhou, 310014 China; 3 National R&D Branch Center for Pelagic Aquatic Products Processing (Hangzhou), Hangzhou, 310014 China; 4 Collaborative Innovation Center of Seafood Deep Processing, Dalian Polytechnic University, Dalian, 116034 China; 5 Sericultural and Tea Institute, Zhejiang Academy of Agricultural Sciences, Hangzhou, 310021 Zhejiang China; 6 Food and Pharmacy College, Zhejiang Ocean University, Zhoushan, Zhejiang 316000 People’s Republic of China

**Keywords:** Gene regulation in immune cells, Diseases

## Abstract

This study aimed to investigate the protective effect of mussel polysaccharide (MP) on cyclophosphamide (Cy)-induced intestinal mucosal immunosuppression and microbial dysbiosis in mice. MP was shown to stimulate secretion of cytokines (SIgA, IL-2, IF-γ, IL-4, IL-10) and production of transcription factors (occludin, claudin-1, ZO-1, mucin-2, IL-2, IF-γ, IL-4, IL-10). Key proteins (*p*-IκB-α, *p*-p65) of the NF-κB pathway were upregulated after MP administration. SCFAs levels, which were decreased after the Cy treatment, were improved after treatment with MP. Furthermore, 16 S rRNA sequencing data of fecal samples revealed, through α-diversity and β-diversity analysis, that MP improved microbial community diversity and modulate the overall composition of gut microbiota. Taxonomic composition analysis showed that MP increased the abundance of probiotics species (*Lactobacillus*) and decreased the proportion of pathogenic species (*Desulfovibrio*). These findings suggested that MP has a potential immunomodulatory activity on the immunosuppressive mice.

## Introduction

Cyclophosphamide (Cy) is a well-known and potent therapeutic drug^1^ with anticancer activity, but its toxic side effects cannot be disregarded^2^. Previous studies have shown that high doses of Cy can damage the gastrointestinal mucosa and induce changes in the composition of the intestinal microbiota, which can then lead to translocation of pathogenic bacteria to vital organs of the body^3^. Immunosuppression is easier to develop in this situation^4^. Inflammation is at the basis of the pathological process of many diseases. Inflammation is a complex but normal physiological response to injury. On the other hand, an uncontrolled inflammatory response is harmful to the body. As the inflammatory response progresses, it can cause tissue damage and disease in the host. In fact, in a state of persistent inflammation, the body enters long-term immunosuppression as soon as the acute phase of the inflammatory response fades^5^.

Considering the history of human evolution, the intestinal microbiota has always been considered as closely associated with intestinal health^6^. The intestines provide the resident microbiota with a natural anaerobic environment^7^. When a large number of microorganisms of the intestinal microbiota adhere to the intestinal wall, a natural protection is created to avoid direct contact of the intestinal wall with harmful substances^8^. The intestinal microbiota can directly participate in the process of eliminating pathogenic bacteria^9^. In this situation, dominant symbiotic bacteria in the intestine are at advantage in terms of number and proportion, being thus able to outgrow and suppress the growth of pathogenic bacteria. A healthy intestinal microbiota improves its niche by decomposing short-chain fatty acids (SCFAs) to nourish cells of the intestinal wall, thereby promoting their growth and replacement as well as the secretion of digestive enzymes in the intestinal wall^10^. This process is undoubtedly important for a timely regulation of the immune response and for the elimination of inflammation^11^.

Polysaccharide are macromolecular compounds constituted of a variety of monosaccharides or monosaccharide derivatives, with various biological activities, such as anti-inflammatory^12^, anti-tumoral^5^, anti-oxidative^13^, anti-viral, and immunity booster^14,15^. Considering the complex structure, the diversity in biological activities and sources, and good application prospects of many polysaccharides, these compounds have been gaining increasing attention. Many studies have shown that polysaccharide have the ability to regulate the intestinal mucosal immunity and are considered potential prebiotics, leading to positive changes in intestinal microbial diversity and composition^16,17^.

Mussels are a type of bivalve mollusks of marine life that grow in the warm offshore environment. Due to their living habits, mussels can be easily collected in the intertidal zone with abundant reefs and destined to human consumption. Mussels are widely consumed in China owing to their high nutritional value^18^. Mussels are also rich in polysaccharides that hold biological activities. Previous research showed that mussels have properties of immune regulation^19,20^, antioxidants^21^, preventing non-alcoholic fatty liver^22^, and reducing cholesterol levels in mice^23^. In view of the positive effects of MP on immunity, inflammatory response, oxidative stress, and other aspects, it can be hypothesized that MP may help alleviate mice in an immunosuppressive state and with poor intestinal health. The use of MP may help to alleviate imbalances in the intestinal microbiota, hence it is of great significance to evaluate the use of MP as an active ingredient in immune regulation^24^. Therefore, this study aimed to further verify the role of MP in the regulation of the intestinal mucosal immunity and microbiota in Cy-treated mice.

## Results

### Effect of MP on body weight and immune organ indexes of mice

**Fig. 1:**
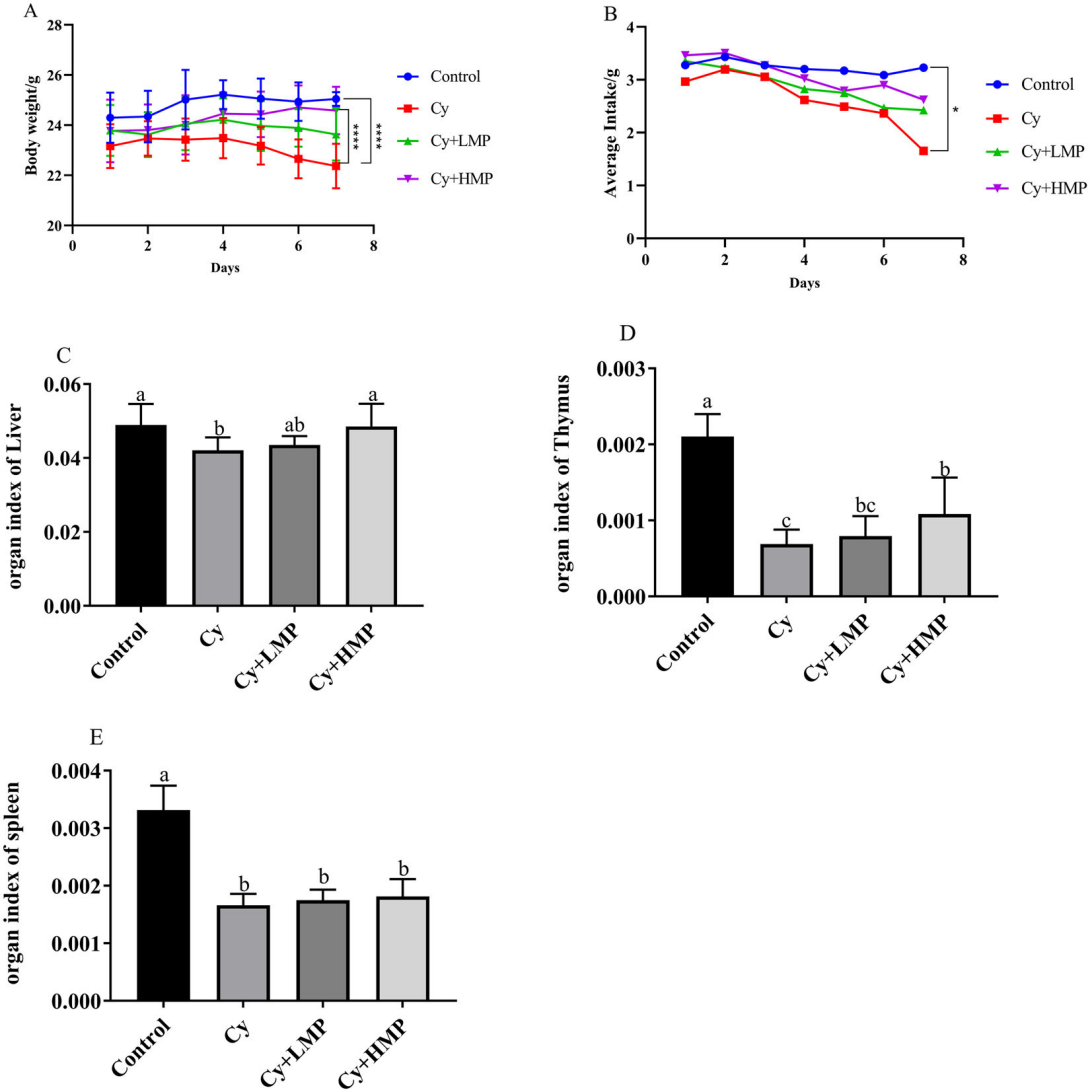
Effect of MP on the Organ index. **A** Body weight, **B** average food intake, **C** Liver index, **D** Thymus Index, and **E** Spleen index. *P < 0.05; ****p < 0.0001. Each column of data marked with a different letter represents a significant difference (*p* < 0.05). Data were ex*p*ressed as mean ± standard deviation.

During the modeling period, body weight of mice in control group was relatively stable, with a trend of slow increase (Fig. 1A). Body weight of the Cy group gradually reduced after Cy administration, while Cy+LMP and Cy+ HMP treatments had a protective effect on weight loss of mice. Average food intake during administration of Cy is shown in Fig. 1B. Control group maintained a steady food intake, while food intake in the Cy group tended to decrease; food intake in Cy+LMP and Cy+HMP groups increased compared with that of the Cy group. Moreover, organ indexes of mice, including liver, thymus, and spleen, which are highly related to immune function, were evaluated. As shown in Fig. 1C–E, compared with the control group, liver, thymus, and spleen indexes of mice treated with Cy decreased significantly (*p* < 0.05). However, these indexes in the Cy+LMP and Cy+ HMP groups were improved, except for the spleen index; there was a slight upward trend in the spleen index after the administration of MP, but which was not significant.

### Effect of MP treatment on the intestinal barrier

**Fig. 2:**
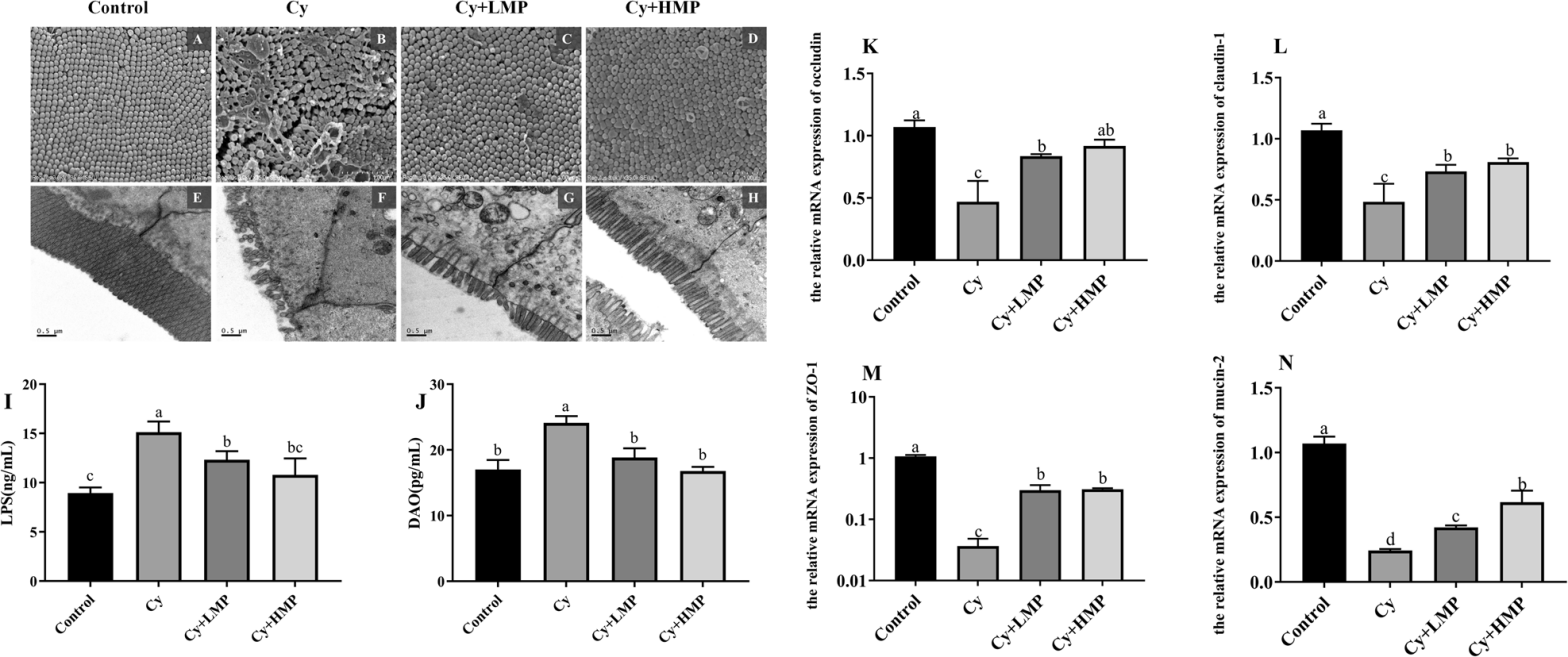
Changes in the villi of ileum after the treatment of MP. (SEM, 35 K×, **A** the control group; **B** the Cy group; **C** Cy+LMP (300 mg/kg); **D** Cy+HMP (600 mg/kg). Effects of epithelial ultrastructure after the treatment of MP (TEM, 20 K×, **E** the control group; **F** the Cy group; **G** Cy+LMP (300 mg/kg); **H** Cy+HMP (600 mg/kg). Effect of MP on the level of (**I**) DAO (**J**)LPS. Effect of MP on the relative expression of intestinal-related genes (**K**) occludin, (**L**) claudin-1, (**M**) ZO-1, (**N**) mucin-2. Each column of data marked with a different letter represents a significant difference (*p* < 0.05). Data were expressed as mean ± standard deviation.

In order to identify mice intestinal mucosal damage, the ileum of mice in each group was evaluated by Scanning Electron Microscope (SEM). As shown in Fig. 2A–D, the microvilli in mice of the control group are regular and well organized. Conversely, microvilli of Cy-treated mice were disrupted, disorganized, and lengths were also inconsistent. In Cy+HMP mice, the brush border of the ileum gradually returned to normal constitution, and the microvilli damaged by Cy were partially recovered. The ileum of mice in each group was also evaluated by Transmission electron microscope (TEM). As shown in Fig. 2E–H, compared with the control group and the MP group, the intestinal cells of the Cy group showed disorganization of microvilli and disappearance of desmosomes. Interestingly, no difference in tight junctions among the four treated groups was found.

Moreover, levels of lipopolysaccharides (LPS) and diamine oxidase (DAO) in the Cy group were significantly higher than in animals of the control group (Fig. 2I, J). When compared with the Cy group, LPS and DAO levels in the Cy+LMP and Cy+HMP groups showed a dose-dependent downward trend. It is well known that the tight junction of the intestine plays a vital role in the normal functioning of the intestinal barrier. As seen in Fig. 2K–N, it can be concluded that Cy greatly inhibits mRNA expression of Zonula occludens-1protein (ZO-1), occludin, claudin-1, and mucin-2 glycoprotein (mucin-2). Surprisingly, after the intervention with MP, this negative effect was abolished. Compared with the control group, the levels of mRNA coding for the above-mentioned proteins in Cy+LMP and Cy+HMP groups were significantly upregulated; in particular, transcripts encoding mucin-2 were significantly enriched in the Cy+HMP group, thus showing that high-dose MP have a certain protective and repairing effect on the intestinal barrier of mice.

### Effect of MP on secretion levels of SIgA

**Fig. 3:**
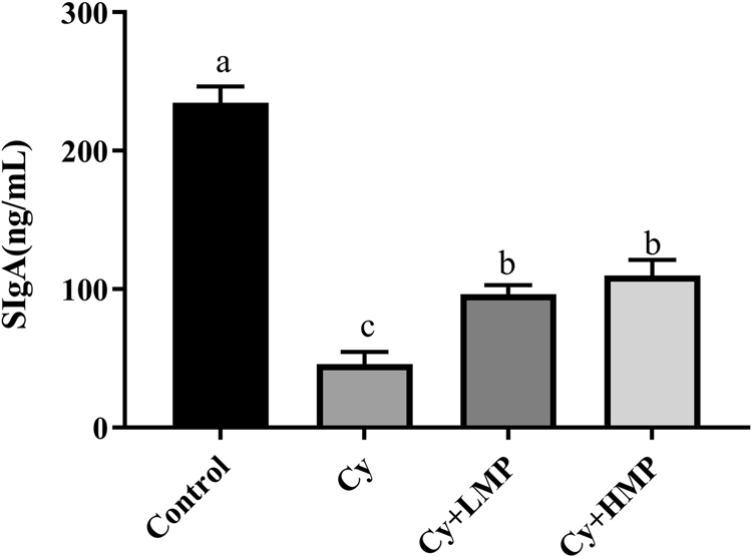
Effect of MP on the levels of SIgA. Each column of data marked with a different letter represents a significant difference (*p* < 0.05). Data were expressed as mean ± standard deviation.

As shown in Fig. 3, when Cy was administered, secretion level of secretory immunoglobulin A (SIgA) significantly reduced, meaning that homeostasis of mice intestinal barrier was disturbed. Compared with the Cy group, MP treatment could significantly increase the secretion of SIgA, even though no significant difference was found between SIgA secretion levels in Cy+LMP group and Cy+HMP group. However, this result still reveals that MP improve the immune function of the intestinal mucosa in mice.

### Effect of MP on the regulation of inflammatory factors

**Fig. 4:**
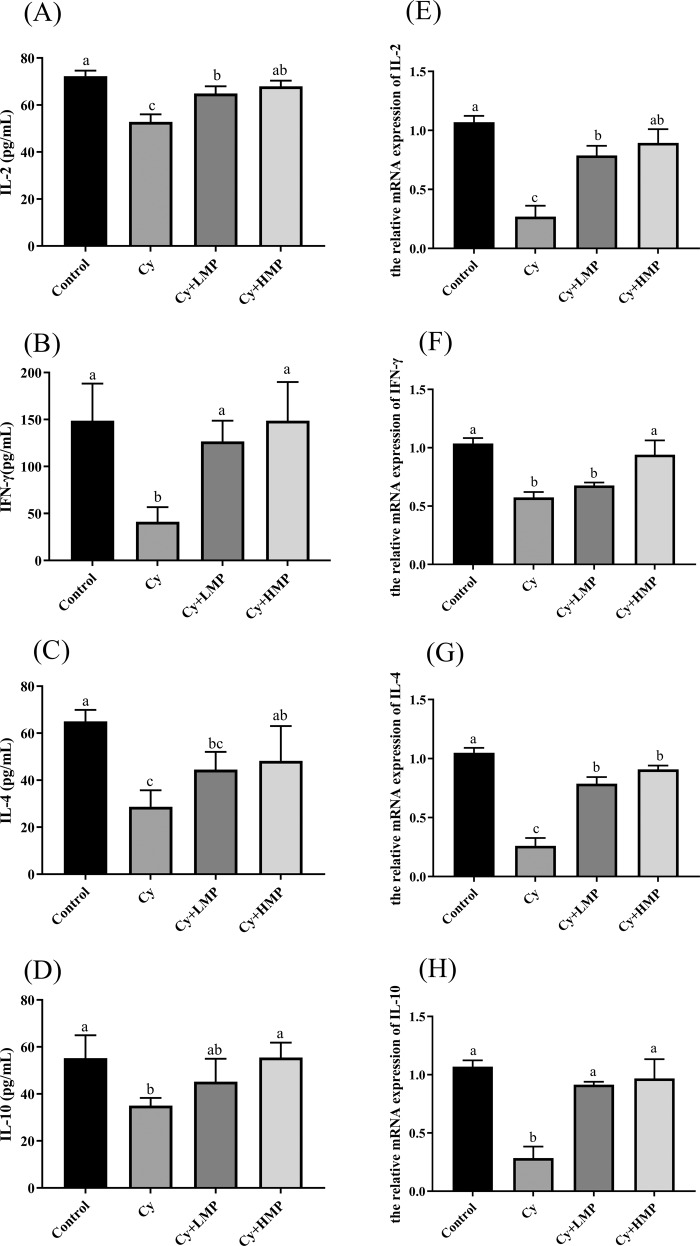
MP down-regulates the secretion level and relative mRNA expression of inflammatory factors. Effects of MP on the secretion levels of (**A**) IL-2, (**B**) INF-γ, (**C**) IL-4, and (**D**) IL-10. Effect of MP on the relative expression of inflammatory genes (**E**) IL-2, (**F**) INF-γ, (**G**) IL-4, and (**H**) IL-10. Each column of data marked with a different letter represents a significant difference (*p* < 0.05). Data were expressed as mean ± standard deviation.

In order to investigate the immunomodulatory effect of MP, secretion levels of T helper type 1 cytokines (Th1) and T helper type 2 cytokines (Th2) were measured by ELISA immunoassay. As shown in Fig. 4A–D, after Cy treatment, secretion levels of Interleukin-2 (IL-2), Interferon γ (IFN-γ), Interleukin-4 (IL-4) and Interleukin-10 (IL-10) in the ileum of mice were significantly reduced. However, after MP treatment, secretion levels of Th1(IL-2, IFN-γ) and Th2(IL-4, IL-10) in ileum of mice increased in a dose-dependent manner. Collectively, these results indicate that MP promoted secretion of Th1 and Th2 cytokines, suggesting that MP have a certain immunomodulatory effect. In addition, in RT-PCR experiments to verify the effect of MP on the expression of cytokines-related genes, compared with the control group, mRNA levels of IL-2, IFN-γ, IL-4 and IL-10 in the Cy group decreased significantly (*p* < 0.05). However, after intervention with MP, mRNA levels significantly increased (*p* < 0.05), especially of IL-2, IFN-γ, IL-4, and IL-10 in the Cy+HMP group (Fig. 4E–H), and their mRNA expression levels were very similar to those in the control group.

### MP regulated the production of key proteins in the NF-κB pathway

**Fig. 5:**
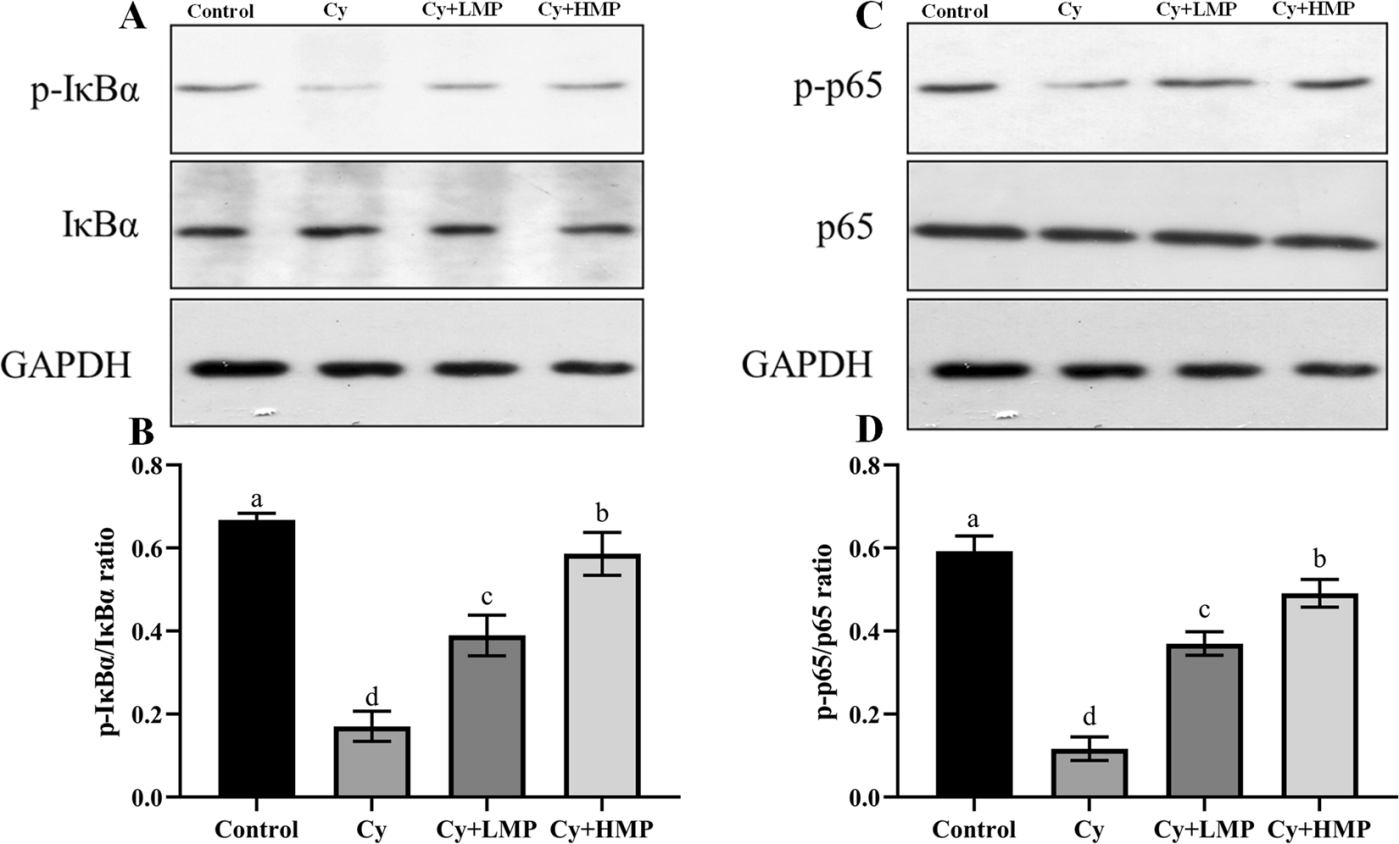
Effect of MP on NF-κB pathway in the ileum of immunosuppressed mice. **A** IκBα and its phosphorylated protein expression levels. **B** The ratio of p-IκBα/IκBα. **C** P65 and its phosphorylated protein expression levels. **D** The ratio of p-p65/p65. Bars with different letters are significantly different (*p* < 0.05). Each column of data marked with a different letter represents a significant difference (*p* < 0.05). Data were expressed as mean ± standard deviation.

The effect of MP on proteins (p65, p-p65, IκBα and p-IκBα) of the nuclear transcription factor kappa B (NF-κB) pathway in the ileum of mice was evaluated by Western blotting. As shown in Fig. 5, Cy inhibited the NF-κB pathway, and levels of p-IκBα and p-p65 in the Cy group were significantly reduced (*p* < 0.05). Intervention with MP reversed protein expression level in the NF-κB pathway and significantly increased phosphorylation of IκBα and p65 in a dose-dependent manner. Based on this result, MP exerted an immunomodulatory effect on Cy-induced immunosuppression in mice by regulating the activity of proteins in the NF-κB signaling pathway.

### Effect of MP on the structure of the gut microbiota of mice

**Fig. 6:**
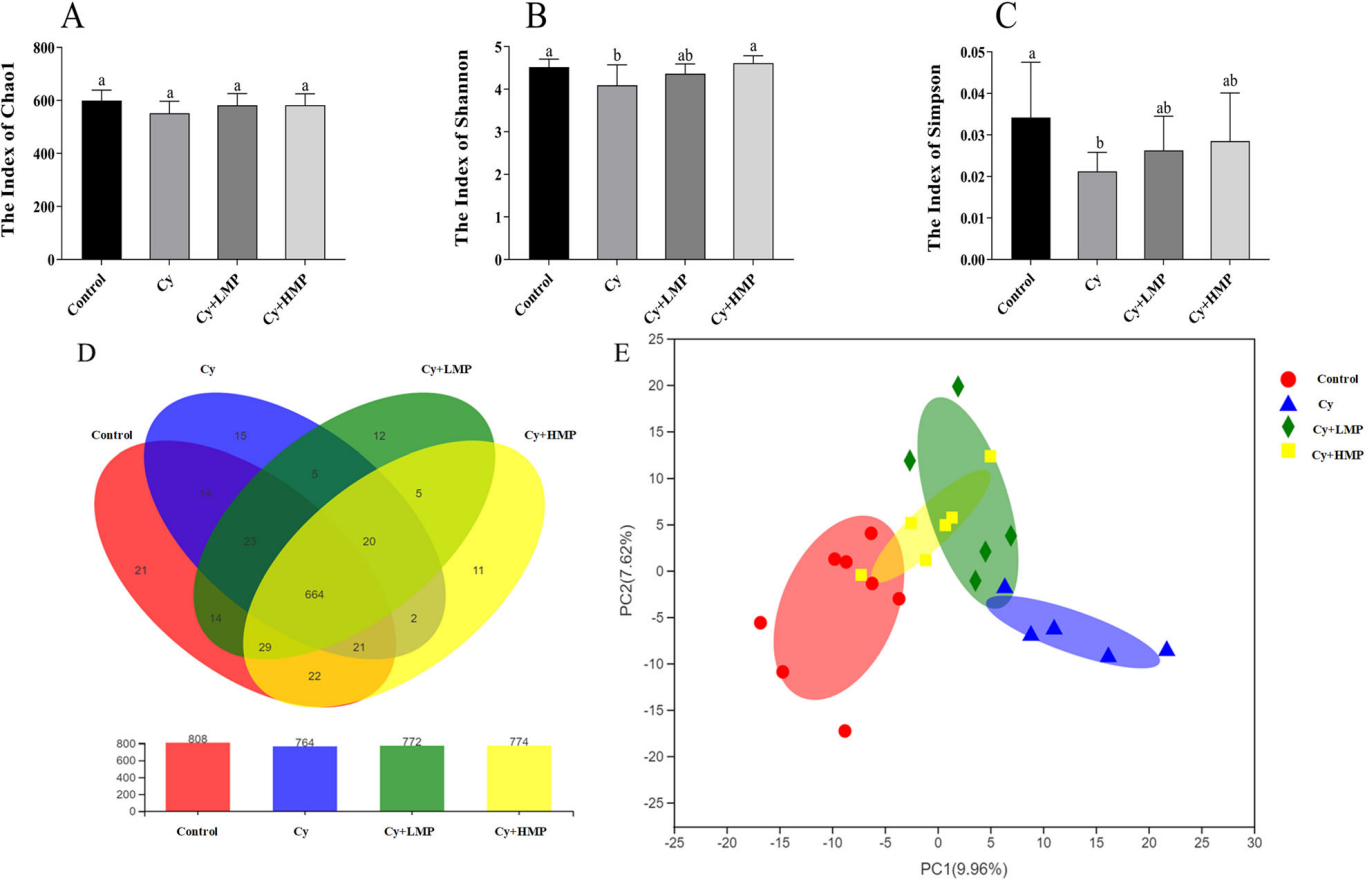
MP alters the composition of the intestinal flora. **A** Chao1, **B** Shannon, **C** Simpson, **D** Veen, and **E** PCA. Each column of data marked with a different letter represents a significant difference (*p* < 0.05). Data were expressed as mean ± standard deviation.

Alpha diversity reflects the abundance and diversity of microbial communities in the gut. As one of the indicators for evaluating microbial community richness, chao1 is often used to estimate the number of OTUs in a sample; the larger the chao index, the greater the number of OTUs, indicating more diversity of microbial species in a sample. As shown in Fig. 6A, abundance of microbial species in the Cy group decreased after Cy administration; however, after administration of MP, the abundance of species increased. Shannon and Simpson indexes are usually used to estimate one of the microbial diversity indexes in a sample. Values of Shannon index showed that the microbial diversity of the Cy group had a downward trend, whereas the microbial diversity improved to a certain extent in Cy+LMP group and Cy+HMP group. In addition, considering Simpson’s diversity index, microbial diversity in the Cy group was also reduced, and that there were positive changes after MP administration, but changes were not significant (Fig. 6B, C).

Through classification, sequences originated from high-throughput sequencing were classified into groups according to their identity, with one group representing an OTU. Bioinformatic and statistical analyses of OTUs at 97% of similarity were performed. Venn diagrams were built to illustrate the number of common and unique species (such as OTUs) in multiple groups or samples, thus more intuitively displaying differences or similarities in samples in terms of composition of gut microbiota. As seen in Fig. 6D, 808 microbial species were found in the control group and 764 microbial species in the Cy group. The number of microbial species in the Cy+LMP group and Cy+HMP group was similar, namely 772 and 774, respectively. There were 664 microbial species shared among the four groups evaluated in this study.

Beta diversity analysis explores the similarity or dissimilarity of microbial communities between different groups of samples. Therefore, Principal Component Analysis (PCA) analysis was performed with the aim to use dimensionality reduction to reveal patterns hidden behind complex data. As shown in Fig. 6E, data representing the control group and Cy group remain largely separated, indicating that composition of the microbial community in these samples is highly dissimilar. Conversely, data concerning the Cy+LMP group and Cy+HMP group overlapped the control group and Cy group. More specifically, the Cy+HMP group partially overlapped the Control group, indicating that positive changes in microbial community species composition were observed in the Cy+HMP group. Based on the previous data results, we uniformly used the data of the Cy+HMP group with better effect for further analysis.

### Effect of MP on microbial community composition

**Fig. 7:**
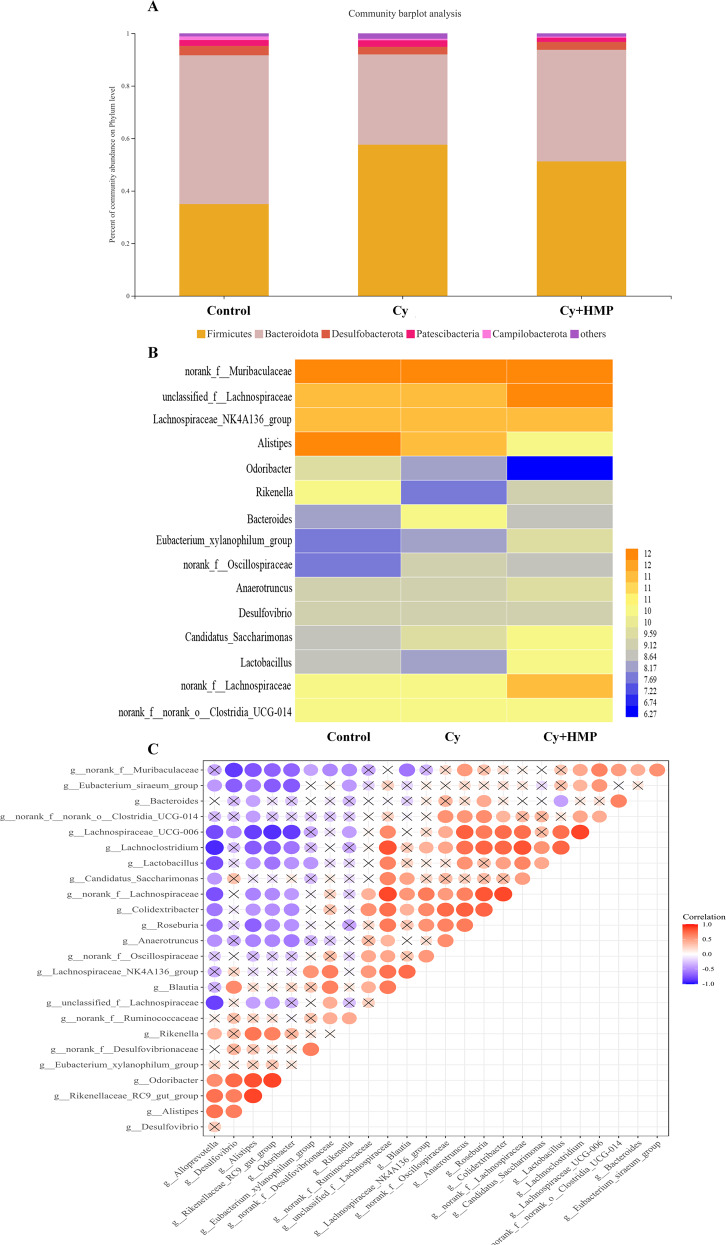
MP promotes the balance of intestinal flora. **A** phylum levels. **B** genera levels. **C** Heatmap diagram of single factor species correlation, Red represents positive correlation, blue represents negative correlation.

Considering the taxonomic classification of the microbial community present in the cecal content of mice, *Firmicutes* and *Bacteroidetes* were the two most abundant phyla among the four groups of samples. As shown in Fig. 7A, compared with the control group, the abundance of *Firmicutes* in the Cy group increased, while the abundance of *Bacteroidetes* progressively decreased in the Cy group, resulting in an increase in the ratio of *Firmicutes*/*Bacteroidetes*, which could pose a threat to the gut health. Conversely, mice treated with high-dose MP reversed the tendency of decrease in the abundance of *Firmicutes*, although not statistically significant. Overall, the abundance of *Bacteroidetes* in the Cy+HMP group also increased.

To identify differences in the bacterial composition among the samples, the relative abundance of the top 15 microbial genera dominant in the samples was analyzed (Fig. 7B). Compared with the control group, the abundance of *Lactobacillus* in the Cy group was reduced, and the proportion of intestinal *Lactobacillus* in MP-treated mice was significantly increased. Moreover, the abundance of *Desulfovibrio* was higher in Cy-treated mice, and intervention with MP reversed this trend. The abundance of *Lachnospiraceae_NK4A136_group* and *Rikenella* was also decreased in the Cy group; however, after intervention with high-dose MP, levels of both microorganisms were reestablished to those found in the control group.

Single-factor network heatmap analysis can reveal the coexistence of microbial species in samples, determine the interaction of species within the same sample, and further explain phenotypic differences in microbiota composition between samples. As shown in Fig. 7C, there is a negative correlation between the abundance of *Lactobacillus* and *Desulfovibrio*, which suggest competition and inhibition between these species. In addition, levels of *Lachnospiraceae_NK4A136_group* and *Lactobacillus* were positively correlated, indicating that these coexist in symbiosis in the intestine of mice.

**Fig. 8:**
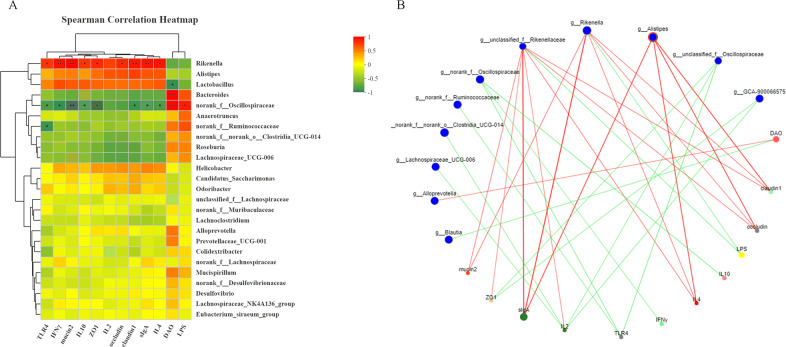
Associations between gut microbiota and immune parameters. **A** Spearman’s correlations heatmap. *p < 0.05; **p < 0.01; **B** network analysis. Red: positive correlation; green: negative correlation.

Spearman’s correlation analysis was used to analyze the relationship between dominant genera and immune response parameters. As shown in Fig. 8A, B, a certain degree of correlation among the intestinal microbiota and immune-related indicators was found. The abundance of *Alistipes*, *Lactobacillus*, and *Rikenella* was negatively correlated with the secretion levels of DAO and LPS, but positively correlated with the secretion of SIgA and with the mRNA levels of IL-2, IFN-γ, IL-4, ZO −1, occludin, claudin-1, mucin-2. In addition, there was a positive correlation between abundance of *Desulfovibrio* and DAO secretion levels.

### Effect of MP on intestinal microbiota composition and levels of SCFAs

**Fig. 9:**
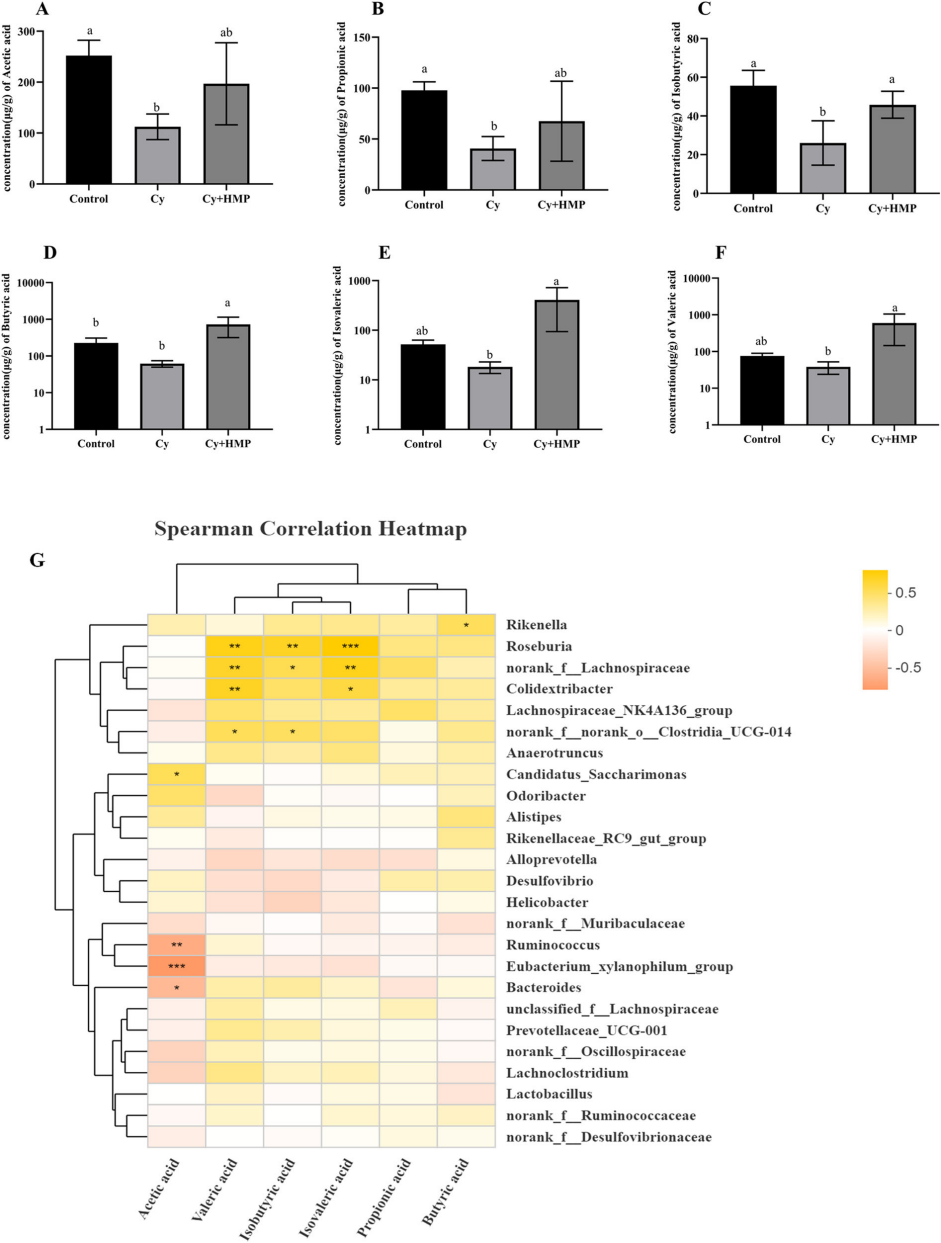
MP increases the content of short-chain fatty acids. **A** Acetic acid, **B** Propionic acid, **C** Isobutyric acid, **D** Butyric acid, **E** Isovaleric acid, and **F** Valeric acid. **G** Spearman’s correlations heatmap between bacterial genus and short-chain fatty acids. Each column of data marked with a different letter represents a significant difference (*p* < 0.05); Different colors of squares represent different *r*-values of Spearman’s correlation. *p < 0.05; **p < 0.01; ***p < 0.001. Data were expressed as mean ± standard deviation.

As shown in Fig. 9A–F, a total of six SCFAs were identified in mice fecal samples, namely acetic acid, propionic acid, isobutyric acid, butyric acid, isovaleric acid, and valeric acid. The content of SCFAs showed a downward trend after administration of Cy. In the Cy+HMP group, the content of SCFAs showed an increasing trend. Moreover, Fig. 9G gives us the following information, *Lachnospiraceae_NK4A136_group* was positively correlated with the secretion of propionic acid, isobutyric acid, butyric acid, isovaleric acid, and valeric acid. *Rikenella* was positively correlated with the six SCFAs detected. However, *Desulfovibrio* was found to be correlated with inhibited secretion of isobutyric acid, isovaleric acid, and valeric acid. Except for butyric acid, the presence of *Lactobacillus* in the intestinal microbiota had a promoting effect on the secretion of the other five acids.

## Discussion

The intestine is an important digestive organ where 60–70% of the body’s immune cells are concentrated^25,26^. Therefore, the intestine can be considered the main battlefield of the body’s immune system^27^. Increased intestinal permeability is potential the underlying cause of many health disorders^28^. In other words, if tight junctions between intestinal cells are compromised, they cannot function as an effective barrier^29^. When foreign substances enter the body, the immune system produce a series of responses to attack and remove harmful substances, thus generating an inflammatory response which further damages the intestinal wall, forming a malignant closed loop^30^. Cy was approved by the FDA as an anticancer drug in 1954. As the most commonly used alkylating drug in the treatment of malignant tumors, Cy can destroy the DNA structure of cancer cells to prevent their replication, leading to cell death. However, severe toxic and side effects may cause damage to the intestinal barrier^3^. It has been demonstrated that longan pulp polysaccharides can reduce damage to the intestinal mucosa by increasing the expression of ZO-1, claudin-1, claudin-4, and mucin-2 in Cy-treated mice^31^, an evidence that is consistent with the findings of the present study. In fact, when MP were used for intervention, mRNA levels of intestinal tight junction proteins ZO-1, occludin, claudin-1, and intestinal mucin-2 increased to varying degrees, indicating that MP can repair intestinal damage caused by immunosuppression induced by Cy.

The body’s first line of defense against diseases is the mucosa^32^. SIgA can entrap pathogens in the mucosal layer through spatial conformation agglutination and bind to specific binding sites on the surface of microorganisms, inhibit their movement, and make them lose their adhesion ability, thereby protecting the mucosa from further damage^33^. Polysaccharides obtained from kidney beans (*Caulerpa lentillifera*) have been shown to promote the production of SIgA^34^. In the present study, after administration of Cy to mice, the level of SlgA secretion in mice decreased. MP were also found to increase SIgA secretion in mice, at both low and high dose. Changes in the organ index indicate that the body’s immune function might be compromised. Studies have shown that polysaccharide obtained from *Tremella* spp. can significantly increase thymus and spleen indexes^35^, while high-dose polysaccharides from *Codonopsis pilosula* can significantly increase spleen, thymus and liver indexes of Cy-induced immunosuppressed mice^14^. In the present study, compared with the untreated group, organ indexes of the Cy-treated group were significantly decreased, while the intervention with MP at different concentrations reversed this phenomenon, suggesting that MP has a potential immune-enhancing effect.

IFN-γ and IL-2 are biomarkers secreted by Th1 cells. IL-4 and IL-10 are biomarkers of Th2 secretion^36^. Secretion levels of the above-mentioned cytokines are closely related to Th1/Th2 balance, which plays an important role in the development of inflammatory diseases^37^. Cy can induce immune function disorder in intestinal cells and cause Th1/Th2 imbalance. A previous study found that the fucoidan fraction from the sporophyll of *Undaria pinnatifida* increased mRNA levels of IFN-γ, IL-2, and IL-4 cytokines, and improved the humoral and cellular immune responses in a Cy-induced immunosuppressed mice model^38^. Compared with mice treated with Cy only, polysaccharide from *C. pilosula* significantly increased the ratio of IFN-γ/IL-4 in the intestinal mucosa, and led to a significant increase in the expression of IL-10 in the ileum of mice^39^. In another study, a water-soluble polysaccharide from the marine green algae *Ulva pertusa* was shown to restore the secretion levels of IFN-γ and IL-10, and regulate the immune function of immunosuppressed mice^40^. In the present study, as a marine animal-derived polysaccharide, MP significantly increased the levels of IFN-γ, IL-2, IL-4, and IL-10 in the ileum of mice immunosuppressed by Cy. MP treatment was also shown to significantly increase the relative mRNA levels of IFN-γ and IL-4. Collectively, these results suggest that MP can restore the balance of Th1/Th2 in mice.

Nuclear transcription factor kappa B (NF-κB) is an important transcriptional regulator, usually in the form of p50-p65 heterodimer combined with its inhibitory protein IκB presented in an inactive state. NF-κB plays a very important role in maintaining the normal physiological functions of the body^41^. Certain external stimuli can activate NF-κB to induce the expression of a variety of genes and the production of a variety of cytokines to participate in the regulation of the body’s inflammation response^42^. Cy acts on the intestinal mucosa causing damage, affecting the secretion of intestinal cytokines, and regulating signaling pathways such as TLRs and NF-κB. Moreover, marine animal-derived polysaccharides have been shown to directly activate NF-κB p65 signaling pathways to regulate the immune system^43^. Polysaccharides of *Cordyceps* spp. have been shown to exert immunomodulatory activity through the NF-κB signaling pathway, and play an important role in the protection of immunosuppressed mice^3^. From the findings obtained in the present study, MP have been shown to significantly enhance the expression of p65 and IκBα phosphorylated protein, indicating that MP may activate the NF-κB signaling pathway in Cy-treated mice and enhance immunity function.

The intestinal microbiota is known to play a key role in the regulation host’s immune function and metabolic processes^44^. Many studies have shown that marine animal-derived polysaccharides have a positive effect on maintaining the balance of the intestinal microbiota of mice^45^. Our experiments showed that the relative abundance of *Firmicutes* in mice treated with Cy increased, while the relative abundance of *Bacteroidetes* was significantly reduced compared to the control group. After treatment with MP, the abundance of *Firmicutes* decreased, although not significantly, and there was also a small increase in the abundance of *Bacteroidetes*. *Lactobacillus* has strong adhesion to the intestinal mucosa and can improve the spatial distribution of individual of the intestinal microbiota. Moreover, *Lactobacillus* can ferment sugars in the intestine to produce lactic acid and antagonize the colonization of the intestine by harmful bacteria. *Desulfovibrio* is a major member of the phylum Proteobacteria, which causes damage to the intestinal barrier by producing sulfide^16^. At the genus level, comparing the abundance between the two species in the Cy group, after Cy treatment, the intestinal microbiota was severely imbalanced, with the abundance of *Lactobacillus* decreased and increase in the abundance of *Desulfovibrio*, hence it can be inferred that inflammation occurred in the intestines of mice in the Cy group. However, the Cy+HMP group can reverse this unfavorable situation. In addition, correlation analysis showed that indicators related to immune globulins and intestinal tight junctions were improved after treatment with MP, thus proving that the changes in the microbiota composition had a positive effect on the intestines of mice. SCFAs can be used as a source of energy for the intestinal cells and improve overall health of intestinal epithelial cells; SCFAs also have unique immune boosting, anti-inflammatory, and other physiological activities^46^. Cui et al. found that a polysaccharide (CSP-1) derived from the marine animal *Cereus sinensis* could significantly increase the thymus and spleen indexes and total SCFAs levels in mice^47^. Similarly, our research also found that MP can promote SCFAs secretion and also had a positive effect on organ index. MP can therefore be used as a prebiotic, which can increase the abundance of beneficial bacteria in the intestine, dislodge harmful bacteria, and increase the level of microbial metabolites such as SCFAs^48^.

This study revealed that MP could protect immune organs by increasing immune organ indexes and relieving immune organ damage; by significantly increasing the serum levels of IL-2, INF-γ, IL-4, IL-10, and SIgA, and by reducing the secretion level of LPS and DAO. MP could also effectively regulate the expression of immunity-related genes by increasing the relative expression of IL-2, INF-γ, IL-4, and IL-10. MP were also shown to improve immunity by regulating the NF-κB signaling pathway and adjusting the composition of the intestinal microbiota towards a more beneficial state to support better overall intestinal health, i.e., by increasing the abundance of *Lactobacillus* and decreasing the abundance of *Desulphovibrio* in the intestine of mice. Moreover, MP also increased the content of beneficial SCFAs in the gut. Overall, MP had a demonstrated positive effect on preventing or ameliorating immunosuppression in mice.

## Methods

Cy was purchased from Sigma-Aldrich (St. Louis, MO, USA). ELISA kits for identification of secretory immunoglobulin A (SIgA) and lipopolysaccharides (LPS) were purchased from Huamei Biological Engineering Co., Ltd. (Wuhan, China). ELISA kit for identification of diamine oxidase (DAO) was purchased from Nanjing SenBeiJia Biological Technology Co., Ltd. (Nanjing, China). ELISA kits for quantification of Interferon γ (IFN-γ), Interleukin-2 (IL-2), Interleukin-4 (IL-4), and Interleukin-10 (IL-10) were purchased from Boshide Bioengineering Limited Company (Wuhan, China). Other chemical reagents were of analytical grade or of the highest grade. MP used in this study was prepared previously by our research group. It had an average molecular weight of 4.25 × 10^3^ Da, which was composed of glucose (0.995), galactose (0.003), glucosamine hydrochloride (0.001), and galactose hydrochloride (0.001).

### Animals and experimental design

Forty male pathogen-free BALB/c mice (five weeks old; 15–18 g body weight) were purchased from Shanghai Laboratory Animal Center (SLACCAS) (Certificate Number: SCXK (hu) 2017-0005; Shanghai, China). Mice were kept at 25 °C. and relative humidity of 50 ± 5%, under a 12-h light-dark cycle, and were freely given standard feed and water. All animal experimental protocols in this study were performed according to guide of the ethical committee for experimental animal care at Zhejiang University of Technology (201907) and were performed in accordance with the Guide for the Care and Use of Laboratory Animals (NIH Publication, ISBN-13: 978-0-309-15400-0, ISBN-10: 0-309-15400-6, 2010).

After one week of adaptation, mice were randomly divided into four groups (*n* = 8), namely: control group; Cyclophosphamide-treated group (Cy group); group receiving low MP concentration with 300 mg/Kg BW/day (Cy+LMP); and group receiving high MP with 600 mg/Kg BW/day (Cy+HMP). Detailed information on animal treatment is shown in Table 1. Mice in the Cy group, Cy+LMP, and Cy+HMP groups were intraperitoneally injected with Cy (50 mg/Kg BW/day) during the last four days (18–21) of life to induce immunosuppression^49^, while mice in the control group received an equal volume of NaCl 0.9%. In this way to build prevention model. On the last day of the experiment, samples were collected after each mouse was weighed. During dissection, liver, thymus, and spleen were collected and their weights recorded to facilitate organ index calculation. Blood was taken from the abdominal aorta. After blood was collected, upper serum was collected by centrifugation to be used in subsequent ELISA experiments. A section of the ileum was collected for ELISA, PCR and Western blotting experiments, and another section was fixed in a centrifuge tube for subsequent scanning electron microscopy (SEM) and transmission electron microscopy (TEM) analysis. The contents of the cecum were collected and stored at −80 °C for 16 S rRNA gene sequencing analysis and determination of short-chain fatty acid contents.

**Table 1:** Experiment grouping and treatment.

Groups	Oral administration	Intraperitoneal injection
	Days1-21	Days18-21
Control	Saline	Saline
Cy	Saline	50 mg Cy /Kg BW/day
Cy+LMP	300 mg MP/Kg BW/day	50 mg Cy /Kg BW/day
Cy+HMP	600 mg MP/Kg BW/day	50 mg Cy /Kg BW/day

### Histopathologic analysis

The ileum was isolated from each mouse and fixed in 2.5% (v/v) glutaraldehyde at 4 °C. Samples were prepared differently according to the standard techniques for SEM and TEM analysis. Samples used for TEM analysis were washed with 0.1 M PBS three times, fixed in 1% osmium acid for 1 h, and rinsed with distilled water. Subsequently, samples were treated with gradient ethanol, dehydrated with pure acetone, embedded in Spurr’s resin overnight, and polymerized at 70 °C. Ileum samples were cut into thin slices and stained with uranyl acetate-lead citrate for TEM analysis. Samples used in SEM analysis were dehydrated with gradient ethanol, dried at the critical point of liquid CO_2_, and then plated with about 30 nm thick gold on the surface of the ileum sample in an ion sputtering apparatus to be observed in a scanning electron microscope. Results of TEM and SEM analysis were evaluated by the Zhejiang Academy of Agricultural Sciences.

### Determination of SIgA, DAO, and LPS levels

Mice were anesthetized, and blood samples were collected. Blood samples were centrifuged (4 °C, 4000 rpm for 10 min) followed by incubation at 4 °C overnight; thus serum was obtained. Levels of SIgA, DAO and LPS in the serum were measured using the above-mentioned ELISA kit immunoassay according to the manufacturer’s instructions.

### Determination of intestinal cytokines levels

Ileum samples were prepared in ice-cold 0.01 M PBS (10%, w/v) and homogenized with an electric homogenizer. Subsequently, samples were centrifuged at 4000 rpm for 20 min at 4 °C, and the supernatant was collected. Levels of IFN-γ, IL-2, IL-4, IL-10 were measured using the above-mentioned ELISA kit immunoassay following the manufacturer’s instructions.

### Gene expression analysis of signaling chemicals

Briefly, 100 mg of mouse ileum tissue was ground in liquid nitrogen, and 1 mL of lysate RZ (DP419, Tiangen Biochemical Technology Beijing Co., Ltd.), and homogenized. After centrifugation at 4 °C at 12,000 rpm, the supernatant was obtained, and 200 μL of chloroform was added, followed by vigorous homogenization for 15 s. The homogenates were left to stand for 3 min, and then the aqueous phase was removed and transferred to another tube where 0.5 times the volume of absolute ethanol was added, followed by mixing. This mixture was transferred to an adsorption column CR3, followed by centrifugation for 30 s. The liquid was discarded, and then protein-removing solution RD was added to the adsorption column, followed by the addition of rinsing solution RW after a final centrifugation. RNase-free ddH_2_O was used to elute obtained RNA. RNA purity and concentration were assessed in a P300 NanoPhotometer (Implen, Germany). FastKing gDNA one-step method was used to obtain cDNA using first-strand synthesis premix reagent (KR118, Tiangen Biochemical Technology Beijing Co., Ltd.). Quantitative real-time PCR (qRT-PCR) was performed to determine the levels of expression of genes coding for IFN-γ, IL-2, IL-4, IL-10, mucin-2, occludin, and claudin-1. SYBR Green FastKing One-Step RT-qPCR Kit (FP313, Tiangen Biochemical Technology Beijing Co., Ltd.) was used in the reactions. qRT-PCR was performed using the Applied Biosystems ViiA™ 7 Real-time PCR system (Thermo Fisher Scientific, NY, USA). β-actin was set as the reference gene for normalizing the expression of target genes. Oligonucleotide sequences are shown in Table 2^50^.

**Table 2:** Primers of RT-PCR.

Gene	Accession number	Primer sequence 5′-3′	Product size(bp)
IFN-γ	NM_008337.4	F: CGGCACAGTCATTGAAAGCC R: TGTCACCATCCTTTTGCCAGT	119
IL-2	NM_008366.3	F: CTCTGCGGCATGTTCTGGAT R: AATGTGTTGTCAGAGCCCTTT	118
IL-4	NM_021283.2	F: CCATATCCACGGATGCGACA R: CTGTGGTGTTCTTCGTTGCTG	131
IL-10	NM_010548.2	F: GGTTGCCAAGCCTTATCGGA R: GAGAAATCGATGACAGCGCC	156
Mucin-2	NM_023566.3	F: CCGGATCTATGCCGTTGCTA R: TCCAGGTGGGTATCGAGTGT	126
Occludin	NM_001360539.1	F: TAGGGGCTCGGCAGGCTAT R: CCGATCCATCTTTCTTCGGGT	104
Claudin-1	NM_016674.4	F: CAACCCGAGCCTTGATGGTA R: ACTAATGTCGCCAGACCTGAAA	169
β-actin	NM_007393.5	F: TATAAAACCCGGCGGCGCA R: TCATCCATGGCGAACTGGTG	117

*F* forward; *R* reverse.

### Western blot analysis

Briefly, 100 mg of ileum tissue was homogenized in lysis buffer containing 1 mL of ice-cold RIPA buffer, 10 μL 50-mM phenyl methyl sulfonyl fluoride (PMSF), and 10 μL protease inhibitor cocktail, and lysed for 15 min at 4 °C, followed by centrifugation at 12,000 rpm for 10 min at 4 °C. The supernatant was collected, and proteins were quantified using the BCA protein assay kit (Beyotime, Shanghai, China). Proteins were denatured by boiling in loading buffer with the anionic denaturing detergent sodium dodecyl sulfate at 100 °C for 5 min. Equal amounts of proteins were subjected to 10% SDS-polyacrylamide gel, transferred onto 0.45 µm polyvinylidene difluoride membranes, and blocked with 5% non-fat milk for 1 h at room temperature. Primary antibodies against NF-κB p65, p-p65, IκBα, p-IκBα, and GAPDH were incubated overnight at 4 °C, followed by treatment with a secondary antibody (HRP-conjugated secondary antibody) for 1 h at room temperature. After three consecutive washes, an enhanced chemiluminescence (ECL) reagent kit (Clinx, Shanghai, China) were used for imaging. Proteins were detected in a FluorChem FC3 (ProteinSimple, Santa Clara, CA, USA), and the relative densities were quantitated in an ImageJ software (1.52n, National Institutes of Health, Bethesda, MD, USA). Each experiment was performed three times independently.

### Determination of cecal microbiota by 16 S rRNA sequencing

Cecal content was collected and immediately frozen in liquid nitrogen before storage at −80 °C. Microbial total gDNA was extracted from the cecal content of six randomly selected mice in the control group, Cy group, and Cy+HMP group, using the KAPA HiFi HotStart PCR Kit (KAPA Biosystems, USA) according to the manufacturer’s instructions. For each sample, the V3-V4 variable region of the bacterial 16 S rRNA gene was chosen for PCR amplifications and was sequenced in an Illumina MiSeq platform (Illumina, SD, USA).

Each PCR reaction (25 μL) included 2×KAPA HiFi HotStart Ready Mix, 25 µM of each primer, 30 ng of template DNA, and PCR-grade water. PCR amplification was conducted as follows: pre-denaturation at 98 °C for 3 min; 25 cycles of 95 °C for 30 s; 56 °C for 30 s, and 72 °C for 30 s; final extension at 72 °C for 5 min. Sequence clustering was performed in UPARSE (v.7.0.1090) based on the RDP Classifier (v.2.11). Sequences were divided into operational taxonomic units (OTUs) with 97% similarity in SILVA (v.132). Sequencing and bioinformatics analysis of cecal content were conducted by HangZhou HanTai Gene Co., Ltd. (Hangzhou, China).

### Short-chain fatty acid quantification

SCFAs were extracted by mixing 20 mg of feces with 500 µL internal standard (hexanoic acid-d3, 10 µg/mL). After homogenization and centrifugation (12,000 rpm, 5 min, 4 °C), the supernatant was transferred to an Eppendorf tube with 5% concentrated sulfuric acid at a ratio of 10:1. Subsequently, an equivalent volume of ethyl acetate was added to the mixture. After centrifugation and incubation at 4 °C for 30 min, the supernatant was removed and used in gas chromatography–mass spectrometry (GCMS) analysis. The analysis was performed in an Agilent 7890 A GC oven coupled to an Agilent 5975 C inert mass selective (MS) detector (Agilent Technologies, Santa Clara, CA, USA). SCFAs were identified by retention times of compounds when compared to standard solutions. Standard solutions of acetic acid, propionic acid, isobutyric acid, butyric acid, isovaleric acid, and valeric acid (at 0.01, 0.1, 1, 10, 100, and 1000 µL/mL) and blank solutions were prepared following the samples preparation procedure.

### Statistical analysis

Data were processed with SPSS 25.0 and GraphPad Prism 9.1.1 software. Data were expressed as mean ± standard deviation (SD) and analyzed by one-way analysis of variance (ANOVA). *p*-values < 0.05 were considered statistically significant. Base calling was used to convert high-throughput DNA sequencing image data into sequences. Sequences were loaded into QIIME2 (1.8.0), and primer sequences were removed by Cutadapt (1.9). QIIME (1.9.1) and Fastp (0.19.6) were used to filter noise sequences, repair sequencing errors, eliminate chimeras and redundancy, and yield representative sequences. QIIME2 (1.8.0) was used for species annotation and OTU classification, and β diversity analysis. Mothur (1.30.2) was used to analyze alpha diversity.

## Data Availability

All data generated or analyzed during this study are included in this published article.
